# Attitudes Toward Mobile Apps for Pandemic Research Among Smartphone Users in Germany: National Survey

**DOI:** 10.2196/31857

**Published:** 2022-01-24

**Authors:** Lorina Buhr, Silke Schicktanz, Eike Nordmeyer

**Affiliations:** 1 Department of Medical Ethics and History of Medicine University Medical Center Göttingen Göttingen Germany; 2 Department of Agricultural Economics and Rural Development University of Göttingen Göttingen Germany

**Keywords:** user, pandemic, smartphone apps, mobile apps, telephone-based survey, Germany, data sharing, data donation, ethics, trust, COVID-19, mHealth, mobile applications, digital health, health applications

## Abstract

**Background:**

During the COVID-19 pandemic, but also in the context of previous epidemic diseases, mobile apps for smartphones were developed with different goals and functions, such as digital contact tracing, test management, symptom monitoring, quarantine compliance, and epidemiological and public health research.

**Objective:**

The aim of this study was to explore the potential for the acceptance of research-orientated apps (ROAs) in the German population. To this end, we identified distinctive attitudes toward pandemic apps and data sharing for research purposes among smartphone users in general and with a focus on differences in attitudes between app users and nonusers in particular.

**Methods:**

We conducted a cross-sectional, national, telephone-based survey of 1003 adults in Germany, of which 924 were useable for statistical analysis. The 17-item survey assessed current usage of pandemic apps, motivations for using or not using pandemic apps, trust in app distributors and attitudes toward data handling (data storage and transmission), willingness to share coded data with researchers using a pandemic app, social attitudes toward app use, and demographic and personal characteristics.

**Results:**

A vast majority stated that they used a smartphone (778/924, 84.2%), but less than half of the smartphone users stated that they used a pandemic app (326/778, 41.9%). The study focused on the subsample of smartphone users. Interestingly, when asked about preferred organizations for data storage and app distribution, trust in governmental (federal or state government, regional health office), public-appointed (statutory health insurance), or government-funded organizations (research institutes) was much higher than in private organizations (private research institutions, clinics, health insurances, information technology [IT] companies). Having a university degree significantly (*P*<.001) increased the likelihood of using a pandemic app, while having a migration background significantly (*P*<.001) decreased it. The overwhelming majority (653/778, 83.9%) of smartphone users were willing to provide their app data for state-funded research. Regarding attitudes toward app usage, striking differences between users and nonusers were found. Almost all app users (317/327, 96.9%) stated they would be willing to share data, whereas only 74.3% (336/452) of nonusers supported data sharing via an app. Two-thirds (216/326, 66.3%) of app users fully or rather *agreed* with the statement that using a pandemic app is a social duty, whereas almost the same proportion of nonusers entirely or rather *disagreed* with that statement (273/451, 60.5%).

**Conclusions:**

These findings indicate a high potential for the adoption of ROAs among smartphone users in Germany as long as organizational providers engaged in development, operation, and distribution are state-funded or governmental institutions and transparency about data-using research institutions is provided.

## Introduction

### Background

After the outbreak of the COVID-19 pandemic, various governments and the European Union decided that digital solutions, most notably smartphone apps, should contribute to pandemic management and research [1,2]. Four different digital public health technologies have been described: (1) mobile apps for proximity and contact tracing, (2) mobile and web apps for symptom monitoring, (3) digital tools for quarantine compliance, (4) data analytic tools for flow modelling [3]. In particular, digital contact tracing has then been intensively debated from practical and ethical perspectives [4-9]. Recently, digital options for providing proof of individual immunization or health status, such as the “Digital Green Certificate” proposed by the European Commission [10], and “check-in-apps,” such as the German “Luca-App,” have also been the subject of contentious debate. In contrast, apps with a primary function of transferring or making available digital data to pandemic-related epidemiological and public health research have been far less publicly discussed. Some of these apps can be connected with a wearable device such as a fitness watch or fitness tracker. We call this type of app a research-orientated app (ROA). ROAs promise to provide answers to various research questions in the field of (digital) epidemiology and public health research. The German Data Donation App [11] is a classic example of an ROA-type pandemic app. From an ethical and social perspective, however, various issues need to be addressed: the consent to collect and the protection of sensitive data (eg, indicating bodily activity and movement); governance structures for data sharing and usage; and public support or even social obligations for such digital health technologies in pandemic research [8,12]. States that have comparatively strict data protection laws and those that are defending permissive standards regarding citizens’ rights such as China, South Korea, or Israel have to find different solutions for consent and voluntary data sharing, whether for pandemic management or research.

### Previous Work

In recent years, there has been an increasing amount of qualitative, quantitative, and mixed methods studies in an international context that have explored behaviors, motivations, and perceptions about mobile phone–based apps for health. They focus on health apps in general [13,14] or on health apps for a specific field of disease (eg, chronic diseases) [15,16]. As pandemics apps are a type of health app, studies on pandemic apps can be considered a further type of domain-specific health app. Since the start of the COVID-19 pandemic, most research on pandemic apps has been carried out on contact tracing apps, mainly focusing on perceptions toward digital contact tracing apps as well as motivations, acceptability, drivers, and barriers for app uptake [17-23]. Most of these studies have focused on privacy and surveillance concerns, including questions of trust and mistrust in different app providers. In most cases, these are national studies conducted as online panels. An exception is the Ipsos Mori survey in the United Kingdom, which was also telephone-based [24]. There are already some cross-national studies that have surveyed the conditions for acceptance of contact tracing apps [25-30]. In the German-speaking context, the studies by Becker et al [20], Kaspar [31], Buder et al [32], and the eGovernment Monitor [33] should be highlighted, since they were published before our data collection started and gave input for questionnaire construction in this study. Studies in the German-speaking context that mainly focused on privacy and surveillance concerns reported a relatively high rate of people who doubt the fundamental benefit of contact tracing apps: around one-third and up to one-half of the respondents were skeptical about using an app [18,33]. Moreover, results differed as to which organizations and providers are trusted in connection with the development and release of smartphone apps and to what extent.

In the context of health apps in general and pandemic apps in particular, current debate is mainly focusing on privacy concerns and perceptions toward sharing health data [13,34]. Beierle et al [35] found that there is a complex picture to describe smartphone users’ willingness to share data with researchers, showing that privacy concerns are not clearly the main factor for not permitting data sharing; personality traits, gender, and age are also considerable factors. Kaspar [31] provided a valuable multiple regression analysis that indicated significant differences in motivations using a contact tracing app or the German Data Donation App (n=406, convenience sample). Interestingly, he found that “motivation for providing the personal data requested by the individual app type was also higher in the case of the contact tracing app (mean 4.48, SD 2.32) compared to the Data Donation app (mean 3.41, SD 2.23; t_405_=10.86, *P*<.001, *d*=0.54)” [31]. Recently, von Wyl et al [22] reported results from their nationwide online survey panel in Switzerland describing differences between users and nonusers of pandemic apps. To the best of our knowledge, there is no study that has systematically analyzed differences between app users and nonusers of pandemic-related apps for German smartphone users with regard to ROA.

### Objectives

This study explored the potential for the adoption of ROA among the German population. We focused on smartphone users and aimed to identify specific challenges for app usage. To our knowledge, this is the first nationwide, telephone-based survey study in Germany since the first pandemic apps (“Corona-Warn-App” and “Data-Donation-Apps”) were released nationally. It is also, to our knowledge, the first study focusing primarily on individual data sharing via smartphone apps for pandemic research. Leading research questions were (1) “Which sociodemographic and personal factors influence the use of a pandemic app among smartphone users in the German population?” and (2) “How do users and nonusers of pandemic apps differ in their motives, attitudes toward pandemic apps, and willingness to share data with researchers?” The objectives of this paper were therefore to identify distinctive attitudes toward pandemic apps and data sharing for research among smartphone users in general and with a focus on differences in attitudes between app users and nonusers in particular. The results can inform empirically based ethical recommendations for the future development, design, and implementation of ROA.

## Methods

### Study Design

We designed a survey comprised of 17 question units (see Multimedia Appendix 1) to explore attitudes toward pandemic apps and toward data sharing for research. The survey study was approved by the local Human Research Review Committee (Reference Number 4/12/20) at the University Medical Center Göttingen.

A representative phone-based survey seemed more appropriate than online panels to reach people who are not internet-savvy or avoid online surveys. Especially when it comes to questions of public acceptance of modern technologies, such as smartphone apps, a broader sampling strategy seemed more appropriate to make statements about the whole population.

Inclusion criteria were people (1) aged ≥18 years, (2) with a registered address in Germany, and (3) who were literate in German. The sample population was comprised of private households in Germany with at least one landline connection and people with at least one mobile phone connection. The population survey was conducted by the company Kantar GmbH. It took approximately 15 minutes to 20 minutes to complete the questionnaire. A dual-frame sampling approach (ie, taking into account both landline and mobile phone numbers) was used. The landline (n=703) and cell phone (n=300) samples were then combined by statistical weighting according to the demographic statistics of the German population (see Multimedia Appendix 2). The survey was conducted anonymously, hence no identifying data were included in the data file Kantar GmbH sent to the authors. Due to incomplete data, 79 cases were excluded from the sample. Thus, our population sample included 924 cases, which is also the number of complete interviews.

### Sample

A representative telephone-based population survey with 1003 people in Germany aged 18 years or older was conducted between December 10, 2020 and January 18, 2021. A sample size of 1000 interviews was originally planned; 3 further interviews were conducted due to already arranged appointments with target persons. The German population aged ≥18 years currently is around 69.4 million. In current survey research, 1000 respondents have proven to be a practicable and statistically acceptable sample size for representative population surveys in Germany. We can refer to the seminal national survey study by Richter et al [36] on acceptability of data donation in medical and health contexts in Germany, which also included a similar sample size (n=1006) using the population-representative survey panel “forsa.Omninet” by the German Forsa Institute [37]. See Multimedia Appendix 3.

### Survey Items

The survey instrument for the phone questionnaire contained closed-ended question types. The questionnaire encompassed 17 question units in the German language and entailed the following domains: (1) current usage of smartphone and pandemic apps, (2) motivations for using or not using pandemic apps, (3) trust in app distributors and data storage, (4) willingness to share coded data with research institutions using a pandemic app and attitudes toward data handling, (5) social attitude toward app use, and (6) demographic and personal characteristics (Multimedia Appendix 1). The composition was informed by an analysis of then-existing surveys about pandemic apps and of another survey we conducted in 2020 on attitudes toward data sharing of wearable data among cardiac patients (publication in preparation). “Pandemic apps” were defined in this survey as native mobile applications for smartphones specifically designed for the containment, management, and research of epidemic and pandemic infectious diseases. Since a pilot test showed that there was no broad understanding of different types of app construction—standalone apps and web apps—we limited the definition to standalone smartphone apps (cf, a study on pandemic web apps by Scherr et al [38]). Multiple answers were possible for 6 question units, 5 questions were formulated as yes/no questions (“yes,” “no,” “I don't know,” “other reason”), and 2 items contained a Likert scale: 1 item with a 4-point Likert scale and the other with a 5-point Likert scale. If a response other than the given answer choices was given, this was recorded unaided by the interviewers. Questions were presented to each participant in the same order; however, the order of within-item responses was randomly assigned to reduce response-set bias. Kantar GmbH was responsible for the implementation of the questionnaire for fieldwork (eg, programming, codes, filter guidance) and pretesting with regard to understandability.

### Statistical Analysis

Descriptive statistics were calculated for all items. Since we were interested in pandemic app usage and attitudes toward data sharing with research institutions, we focused on the subsample of smartphone users in our statistical analysis (n=778). Chi-squared tests were used to identify differences between users and nonusers of the app regarding the willingness for data sharing, social attitudes toward app usage, perceptions of the trustworthiness of the app provider, and the preferred location for data storage. To quantify the factors influencing app usage, logistic regression analysis was used. For this purpose, sociodemographic variables such as age, gender, education, place of residence, and migration background were included in the model. In addition, personal experience of being directly infected or knowing someone who has been infected by the COVID-19 virus was elicited (personal affection). Statistical significance was determined by *P* values <.05. Likert-scale answers were pooled into categories (eg, “fully agree” and “rather agree” into “fully/rather agree” and “entirely not agree” and “rather not agree” into “entirely not/rather not agree”). All statistical analyses were carried out using SPSS version 26 (IBM Corp, Armonk, NY). Due to the fact that we used weighted data, the sample size may differ by ±1 in some analyses due to rounding effects. Calculating with weighted data also has the effect that percentages can deviate minimally in the decimal place compared with the quotient n/N in natural numbers. We marked all cases in which this deviation occurred with an asterisk (*) or respectively, a reference mark in tables. For a detailed description of the weighting, see Multimedia Appendix 2. Logistic regression analysis was conducted, as the statistical assumptions were met, and all rating scales were treated as nominal (except gender as an ordinal variable), including the dependent variables. Ordinal regression models, however, could be considered as an alternative approach (with distinct limitations).

## Results

### Demographic Characteristics of Smartphone Users

Of the 924 participants that were included for statistical analysis, 84.2% (778/924) stated that they used a smartphone. The following analysis refers to this subset of smartphone users, as we deemed smartphone usage a condition for technology-specific considerations on pandemic app usage. We assumed that inclusion of non-smartphone users in the statistical analysis would have engendered a mixed sample of distinctive versus hypothetical usage attitudes.

Table 1 presents the demographic and personal characteristics of the sample of the survey participants and the subsample of smartphone users. Compared with the whole sample, whose demographic characteristics are representative of the German population, smartphone users differed slightly in 3 regards: (1) They were younger, (2) they were more likely to have a higher level of education, and (3) they were personally affected by the COVID-19 pandemic slightly more often (affectedness was reported according to their statements). The mean age of the survey sample was 50.19 (SD 17.96) years, and age ranged from 18 years to 95 years; the mean age of the analytic sample was 46.84 (SD 16.96) years, and age ranged from 18 years to 95 years. A total of 37.2%* (290/778) of the smartphone users indicated that they had an A-level or university degree; this is slightly more than the participant sample of whom only 32.8%* (304/924) reported having an A-level or university degree. In terms of COVID-19 infection, 37.8% (294/778) of the smartphone users reported they had been personally affected, whereas 34.1% (315/924) of survey participants reported being personally affected.

**Table 1:** Sociodemographic profile of survey participants (n=924) and the subsample of smartphone users (n=778).

Characteristic		Survey participants (n=924), n (%)	Smartphone users (n=778), n (%)
**Gender**			
	Female	474 (51.3)	404 (51.9)
	Male	450 (48.7)	374 (48.1)
	Nonbinary	0 (0)	0 (0)
**Age (years)**			
	18-30	153 (16.6)	153 (19.7)
	30-39	120 (13.0)	119 (15.3)
	40-49	175 (18.9)	168 (21.5^a^)
	50-59	183 (19.9^a^)	153 (19.6^a^)
	60-69	128 (13.8^a^)	94 (12.1)
	≥70	165 (17.9)	91 (11.7)
**Education**			
	None/still in school	51 (5.5)	44 (5.7)
	Without A-level	570 (61.7)	444 (57.1)
	A-level	138 (14.9)	133 (17.1)
	Academic degree	166 (17.9^a^)	157 (20.1^a^)
**Immigration background**			
	Yes	170 (18.4)	156 (20.1)
	No	754 (81.6)	622 (79.9^a^)
**Region**			
	West German states	765 (82.8)	645 (83.0^a^)
	East German states (including Berlin)	159 (17.2)	133 (17.0^a^)
**Personally affected by COVID-19 infection or knowing someone who was**			
	Yes	315 (34.1)	294 (37.8)
	No	609 (65.9)	484 (62.2)

^a^Due to the fact that we used weighted data, the sample size may differ by ±1 in some analyses due to rounding effects. Calculating with weighted data also has the effect that percentages can deviate minimally in the decimal place compared with the quotient n/N in natural numbers. For a detailed description of the weighting, see Multimedia Appendix 2.

### Attitudes Among Smartphone Users Toward Pandemic App Providers and Toward Data Sharing With Research Institutes

**Figure 1:**
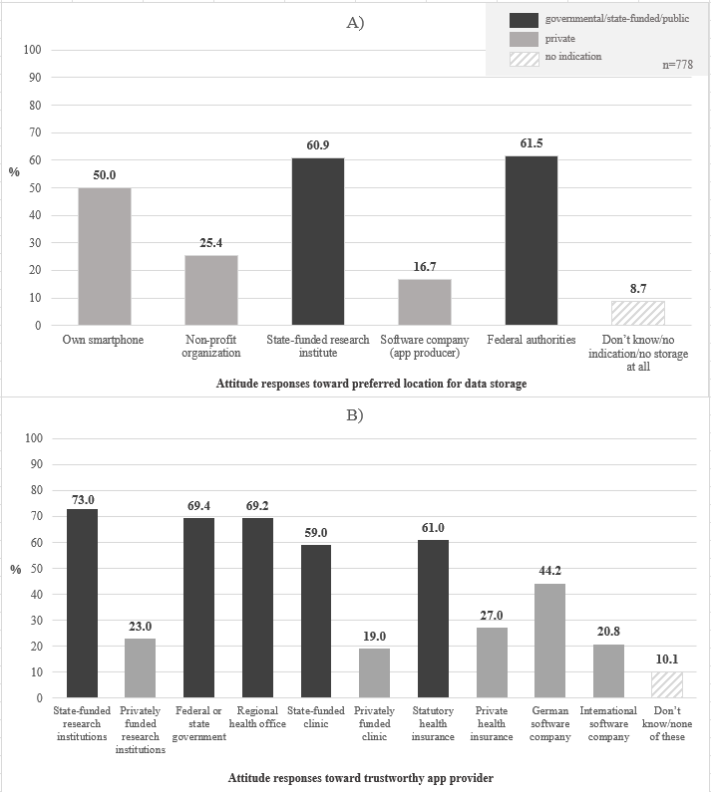
Attitude responses toward (A) preferred location for data storage and (B) trustworthy app provider among smartphone users (n=778).

**Figure 2:**
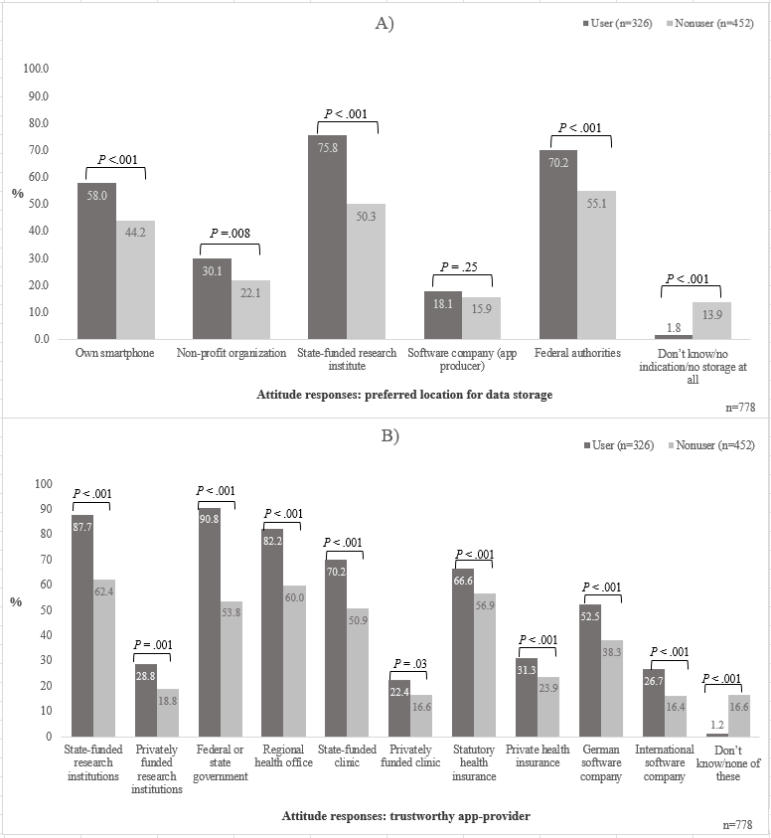
Different attitude responses toward (A) preferred location for data storage and (B) trustworthy app provider among app users (n=326) and nonusers of pandemic apps (n=452).

**Figure 3:**
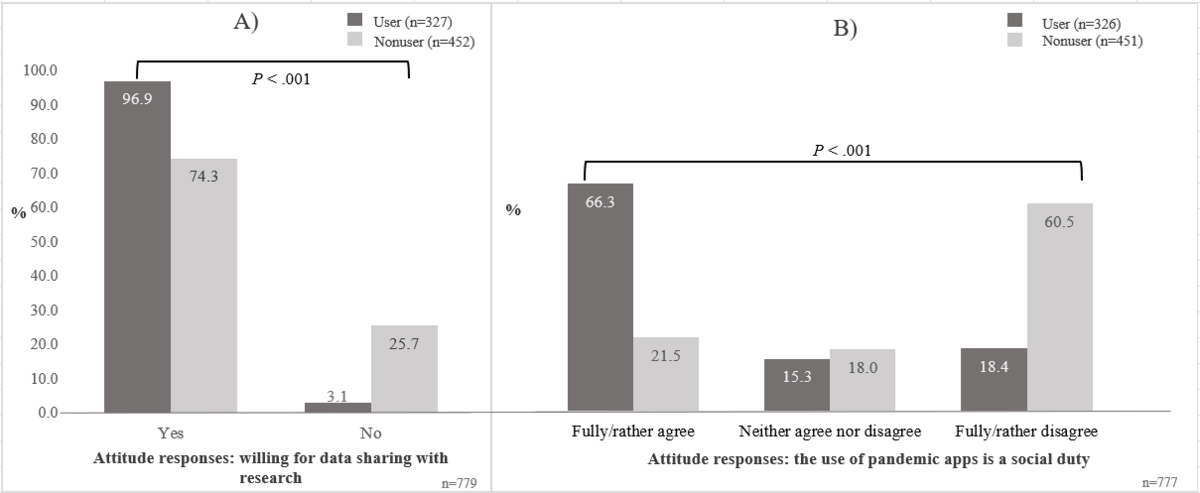
Different attitude responses toward (A) data sharing for research and (B) the statement, "the use of pandemic apps is a social duty" among app users (n=327 and n=326, respectively) and nonusers of pandemic apps (n=452 and n=451, respectively). Due to the fact that we used weighted data, the sample size may differ by ±1 in some analyses due to rounding effects. For a detailed description of the weighting, see Multimedia Appendix 2.

Our analysis of attitudes focused on 2 major topics. First, we report attitudes toward app providers, by which we understand organizations and institutions involved in the development, provision, and operation of pandemic apps (see Figure 1). Second, we present results on attitudes toward sharing data collected by a pandemic app for research (Table 2, Table 3, and Multimedia Appendix 4). The descriptive analysis on attitudes is supplemented by a regression analysis on app usage among smartphone users (Table 4). In a third step, we focused on differences between users and nonusers of pandemic apps on these and related issues (Figures 2 and 3).

**Table 2:** Attitudes among people willing to share data (“data sharers,” n=653) for research via an app among smartphone users (n=778).

Attitude responses among data sharers (n=653)		Results, n (%)
**Kind of data to be shared^a^**		
	Don’t know/none of these	10 (1.5)
	Data collected by a fitness watch	215 (33.0^b^)
	Continuous data (ambient temperature)	298 (45.6)
	Health-related data	330 (50.5)
	Location and movement data	366 (56.1^b^)
	Contacts with other people	436 (66.8)
	Data manually entered in the app	447 (68.5)
	Test results	553 (84.8^b^)
**Preferred way and mode of data transmission to the research institute^a^**		
	Don’t know/none of these	8 (1.2)
	Calling a video hotline of the research institute	58 (8.9)
	Calling a telephone hotline of the research institute	101 (15.5)
	Sending the data via SMS	123 (18.9^b^)
	Sending the data via email	169 (25.9)
	By entering the data on the website of the research institute	207 (31.7)
	Sending the data automatically to the research institute	379 (58.1^b^)
	By enabling data sharing in the app each time	437 (66.9)
**Transparency about data-using research institutes**		
	Not so important/not at all important	158 (24.2)
	Very/rather important	495 (75.8)

^a^Multiple answers were possible.

^b^Due to the fact that we used weighted data, the sample size may differ by ±1 in some analyses due to rounding effects. Calculating with weighted data also has the effect that percentages can deviate minimally in the decimal place compared with the quotient n/N in natural numbers. For a detailed description of the weighting, see Multimedia Appendix 2.

**Table 3:** Attitudes among people not willing to share data (“non-data sharers,” n=125) for research via an app among smartphone users (n=778).

Attitude responses among non-data sharers (n=125)			Results, n (%)
**Why people do not share data^a^**			
	Don’t know	3 (2.2^b^)	
	Other reasons	9 (7.2)	
	I am worried that the data will be leaked.	78 (62.2^b^)	
	I doubt that this data will help research.	78 (62.4)	
	I am concerned about unknown third parties using my data.	85 (68.2^b^)	

^a^Multiple answers were possible.

^b^Due to the fact that we used weighted data, the sample size may differ by ±1 in some analyses due to rounding effects. Calculating with weighted data also has the effect that percentages can deviate minimally in the decimal place compared with the quotient n/N in natural numbers. For a detailed description of the weighting, see Multimedia Appendix 2.

**Table 4:** Multivariable correlates of pandemic app usage.

Variable	Coefficient	*P* value
Constant	–0.564	.02
Male (vs female)	0.069	.65
Age groups	0.055	.27
University degree (vs no university degree)	1.081	<.001
Eastern Germany (vs western Germany)	–0.494	.02
Immigration background (vs no immigration background)	–1.242	<.001
Being affected (vs not personally affected)	0.279	.09

#### Attitudes Toward App Providers: Trust in State (-Funded) Organizations

Our survey revealed that German smartphone users demonstrated a strong preference for state-funded or governmental organizations with regard to storage of app data as well as app providers when asked which providers the participants considered most trustworthy. We considered the statement of preference for a certain app actor for data storage to be an indication of trust in that app actor (or one's smartphone) for data storage and management. As Figure 1A indicates, the majority of smartphone users preferred state-funded research institutes (474/778, 60.9%) or federal authorities (478/778, 61.5%*) when asked where the data for the pandemic app should be stored. It is noteworthy that those rates are even higher than those for using one’s own smartphone as data storage (389/778, 50.0%). Among smartphone users, the least preferred storage location was the software company producing the pandemic app (130/778, 16.7%). This pattern has been consistently mirrored when it comes to trust attitudes toward distributors of pandemic apps. Trust in governmental (federal or state government, regional health office), public-appointed (statutory health insurance), or government-funded research institutes or organizations as app distributors was much higher than trust in private organizations (research institutions, clinics, health insurance). For example, 61.0%* (475/778) of participants considered public health insurance trustworthy distributors for pandemic apps, but only 27.0% (210/778) reported the same for private health insurance.

Interestingly, German software companies were classified twice as trustworthy (344/778, 44.2%) as international companies (161/778, 20.8%*). Figures 1A and 1B illustrate the obvious differences in trust between state(-funded) and private organizations.

#### Factors Influencing the Usage of a Pandemic App

Less than half of participants stated that they used a pandemic app (326/778, 41.9%).

We used logistic regression analysis to determine which sociodemographic characteristics influenced the probability of using or not using a pandemic app. Having a university degree significantly (*P*<.001) increased the likelihood of using a pandemic app, while having a immigration background significantly (*P*<.001) decreased the likelihood of using a pandemic app (see Table 4). Furthermore, residence in the eastern part of Germany reduced the likelihood of using a pandemic app compared with a residence in the western part (*P*=.02; Multimedia Appendix 5). No significant influence was shown for age, gender, or being personally affected by COVID-19 or having relatives or friends who have been infected.

#### Attitudes Toward Data Sharing With Research: High Willingness

The high level of trust in state-funded app providers is matched by the fact that 83.9% (653/778) of smartphone users were willing to provide their app data for state-funded research. We called that subsample of people willing to share data with research institutions “data sharers.” When asked about what kind of data people were willing to share for research, the vast majority indicated test results (553/653, 84.8%*), followed by contact tracing data (436/653, 66.8%). In contrast, only one-third (215/653, 33.0%*) were willing to share data via a pandemic app that were originally collected by a digital mobile device, such as a fitness watch (Table 2, Multimedia Appendix 4). Furthermore, there was a distinct preference for entering data manually into an app (447/653, 68.5%) versus data collected continuously and automatically (298/653, 45.6%). The preferred mode of data transmission, however, was not uniform: Two-thirds (437/653, 66.9%) were in favor of user-initiated data transmission, whereas a large number (379/653, 58.1%*) of respondents affirmed automatic data transmission to a research institute. For one-third of data sharers (207/653, 31.7%), manual entry on a research institute’s website was also an option. Notably, there was a clear trend when it came to the importance of transparency about which research institutions will use the app data. For the vast majority of data sharers (495/653, 75.8%), it was very or rather important that those research institutes were clearly designated (Table 2, Multimedia Appendix 4). Overall, these results indicated a high willingness for data sharing for public research. Interestingly, among the data sharers, a majority expressed a wish for self-controlled data transmission (437/653, 66.9%) and transparency about the involved data-analyzing institutions (495/653, 75.8%).

The 3 most frequent reasons why people were not willing to share their data for research (125/778, 16.1%) were concerns about lack of control of app data (85/125, 68.2%*; ie, the concern that third parties were using the data without consent), that data would be leaked (78/125, 62.2%*), and doubts on whether app data really would bring research forward (78/125, 62.4%; Table 3, Multimedia Appendix 4).

#### Nonusers of Pandemic Apps Have Less Trust in State-Funded Organizations

We found substantial differences in attitudes toward pandemic app providers between users and nonusers of pandemic apps (Figure 2). Nonusers of pandemic apps showed higher approval for public government or state-funded organizations but their trust regarding data storage and distribution of pandemic apps is considerably lower. Figures 2A and 2B provide a comparison of pandemic app users versus nonusers. Although 75.8% (247/326) of pandemic app users preferred state-funded research institutes for data storage, only 50.3% (227/451) of nonusers did so (*P*<.001). Furthermore, 90.8% (296/326) of pandemic app users indicated trust in federal and state governments as app providers, compared with only 53.8% (243/452) of nonusers (*P*<.001). However, no statistically significant differences between users and nonusers of pandemic apps were found regarding software companies as a preferred location for data storage (*P*=.25).

#### Nonusers of Pandemic Apps Are Less Willing to Share Data With Research Institutes

As indicated in Figure 3A, there was high covariance between app usage and the willingness to share data with research institutes via a pandemic app. The overwhelming majority (317/327, 96.9%) of app users stated they would be willing to share data, whereas only 74.3% (336/452) of nonusers supported data sharing via an app; thus, users and nonusers differed significantly in their attitude toward data sharing for state-funded research via an app (*P*<.001). This is consistent with our findings on decreased trust in state-funded research institutes as data storage and app providers (see the previous section). The differences between users and nonusers of pandemic apps were most apparent in the moral and social attitudes toward pandemic app usage (Figure 3B). Two-thirds of app users (216/326, 66.3%) fully or rather *agreed* with the statement that using a pandemic app is a social duty, whereas almost the same proportion of nonusers completely or rather *disagreed* with that statement (273/451, 60.5%). Thus, the moral and social attitude for app usage was inverted between users and nonusers of pandemic apps.

## Discussion

### Principal Findings and Comparison With Prior Work

#### Overview

This study examined pandemic app usage and attitudes toward data sharing with research institutes among a sample of smartphone users, which represented a subsample of a representative population survey in Germany. Our study provides several important findings. In the following sections, we focus on 4 of them. First, the results showed a high willingness to share data with state-funded research institutes for pandemic research, but the willingness for data sharing went along with a strong need for self-controlled data handling and transparency about the involved data-analyzing research institutions. Second, there was a remarkable decline in trust toward private providers and organizations involved in data storage and distributing pandemic apps when compared with state-funded organizations. Third, regression analysis showed app usage is positively correlated with a higher level of education. Fourth, our study revealed significant differences in trust attitudes between app users and nonusers.

#### High Willingness for “Self-Determined” Data Sharing With Research Institutes

One of the aims of this study was to elicit peoples’ attitudes and concerns toward sharing data collected by pandemic apps. The overwhelming support for data sharing via pandemic apps for research purposes among smartphones users in Germany is consistent with previous surveys that examined the willingness for “data sharing” or “data donation” [39]. For example, the online survey panel by tmf/Medical Informatic Initiative and Richter et al [36] indicated a consent rate of 78.8% (n=1006) for “data donation” (ie, a consent-free approach) for medical research among German adults in 2019 (cf, Mello et al [40] for patients’ views on data sharing with research in the United Kingdom). Becker et al [20] showed that a large portion of participants disagreed with providing governmental organizations with anonymous user data to contain the pandemic in Germany. However, the results of the study by Becker et al [20] showed that app adoption was not negatively affected if the data-receiving organizations were public health authorities or research institutes.

In the international context, the picture on the willingness to share data in general is quite heterogeneous. For example, Abeler et al [41] reported that 64.6% of survey participants in the United Kingdom (n=1055) would permit data sharing with researchers after the pandemic. Maytin et al [23] reported for young adults in the United States that 45.1% (231/513) would agree or strongly agree with actively providing health data via an app. A possible explanation for this might be that answers on this topic strongly depend on how the question is posed, how the app providers are presented, and which data participants are asked to share for what purpose.

#### Implications for Policy Makers

When informing policy makers about affirmative attitudes among smartphone users toward willingness to share data with research institutes, those findings should be contextualized with stated preferences for criteria of data sharing and processing. In our study, we explored different criteria that allow for a more nuanced picture: (1) the kind of data to be shared (eg, health data, location data), (2) transparency about data-receiving research institutions, (3) the mode of data collection, and (4) data transmission (Table 2). Our findings indicated that, to gain sufficient rates of people sharing app data for pandemic research purposes, pandemic apps should have the following features: (1) type of data to include test results, contacts with persons, location, and movement data (less support for data from a fitness watch); (2) detailed transparency about data-receiving research institutes (vs no transparency about the data receiver); (3) manually entered data (vs automatically collected data); (4) manually enabled data transmission *or* automatic sending; (5) storage of collected or disclosed data via pandemic app on servers of the respective state-funded research institute (least support for app storage by private organizations such as tech companies).

In summary, we conclude that those willing to share data for research purposes express a strong interest in a self-determined way of data sharing. This means that mechanisms of manual handling such as activation of data transfer, a set of selected kinds of data, and comprehensible and detailed information about data processing would likely increase willingness for data sharing with research institutions. However, since automatic data transmission is also endorsed by a large portion of participants, the picture is more complex. An ambiguous tendency in attitudes toward data transmission was also reported by Becker et al [20]. Hence, an option might be to provide app users with the option to select between automatic and manual data transmission. Further research is then needed to examine users’ long-term satisfaction with these options. The need for more research in this area is also reinforced by recent studies that indicate that the preferred kinds of data willing to be shared may also differ among age groups [23]. Furthermore, the issue of a self-determined manner of data sharing should be examined in relation to “eHealth literacy,” sometimes also called “media health literacy” or “(digital health) data literacy” [42,43]. To measure eHealth literacy in the context of pandemics, the eHealth Literacy Scale (eHEALS) developed by Norman and Skinner [42,44] seems very promising. Future research should test whether eHealth literacy positively correlates with beliefs in the benefits of (pandemic) apps and the willingness to share data with pandemic research (see, for example, the patient survey study by Knitza et al [45] in rheumatology using the validated German version of eHEALS [46]).

#### Gap in Trust Between State-Funded and Private Organizations

One important finding in our regard is the extent to which attitudes toward state-funded and private app providers vary among smartphone users: Private providers were considerably less trusted with data storage and providing an app. Here, we interpreted the preference of a storage location as an expression of trust in this specific organization. We found that trust in state-funded research institutes and governments for the storage of app data is very high (almost two-thirds of smartphone users). This is an encouraging message for state-funded research intuitions even if ongoing public discussions about privacy, data security, governmental surveillance practices, and centralized versus decentralized storage solutions for pandemic apps might give the opposite impression [17,47,48]. However, there is paradox-like situation. On the one hand, current debates on privacy and the willingness of governmental providers to take those concerns into account (eg, as with the German Corona-Warn-App, using open-source code and decentralized storage) might have been seen as trust-building efforts in favor of democratic governments and as a clear demarcation from state surveillance tendencies. On the other hand, increasing awareness and media reports on how information technology (IT) companies use data streams and cloud backups (eg, Apple iCloud or Google Cloud) may have also increased skepticism toward such providers, especially when it comes to public goods, such as health issues. National or cross-country surveys such as those by Simko et al [25], Hargittai et al [28], and Wiertz et al [21] as well as the British IPSOS MORI report [24] support this interpretation. They also report a disparity in terms of trustworthiness between government agencies, health departments, and IT companies, either big tech companies or start-ups. However, there are noticeable national differences. For example, the studies by Wietz et al [21] in the United Kingdom and Hargittai et al [28] in the United States reported a significant difference in public trust between the national government and the top national health authorities (National Health Service in the United Kingdom and Health Protection Agency in the United States), with the latter being significantly more trusted. In contrast, our results cannot confirm this kind of split concerning trust in official health authorities and research institutions in Germany. Although for Kaspar [31], it was still an open question in 2020 “as to whether different providers are assessed as having different levels of trust” [31], our findings provide a clear answer to this question.

#### The Challenge for Public-Private Partnerships for Pandemic Apps

The large gap between state-funded and private providers poses a challenge for the reality of pandemic app development, which is mainly achieved via public-private partnerships. Considering that pandemic research is of extremely high public health relevance and therefore differs from many (not all) other areas of mobile health (mHealth) where health behavior or health research addresses a smaller population of patients, research on pandemic apps can clearly benefit from a strong emphasis on the public partner. However, the high level of trust in government and state-funded research institutes as app providers can be gambled away if there is an increasing reliance on private-public partnerships in which tech companies co-determine the technical and design solutions, as was the case when Apple and Google offered governments their common exposure notification application programming interface (API) [4,49,50]. However, digital contact tracing apps in the COVID-19 pandemic have not yet a reached a sufficient level of broad uptake, such as at least 60% to 70%, which in turn is necessary for validating them as effective tools for pandemic containment and management [51,52]. In our case, 41.9% (326/778) of participants confirmed the use of a pandemic app, which is in accordance with previous studies on adoption rates in 2020 in Germany [33], thus also indicating a plateau in app uptake [53]. But since research via pandemic apps can benefit from a distinctly smaller uptake rate, such as 20% to 40%, a lower app uptake is still productive. However, trustworthiness remains an important component—also when considering future pandemic apps that address more local outbreaks (eg, within hospitals as for multidrug-resistant infections) or for infections among socially vulnerable groups.

#### User Characteristics and Attitudes Toward App Usage

In line with the large longitudinal survey from Munzert et al [53], we found that, in the German context, the level of education, especially in terms of university degree(s), showed a significant impact on the uptake of app usage (*P*<.001) among smartphone users. In contrast, an immigration background significantly decreased the probability for app adoption (*P*<.001). Academic degree has a higher impact than immigration, but we must also consider that both social factors are also interfering. We take this as an indication that culturally formed preferences as well as linguistic aspects of information around apps can be important factors for usage. More research is definitively needed on this subject [54], also to develop culturally sensitive app information for a diverse population. This applies all the more as our findings showed a weak correlation between living in the eastern part of Germany and reluctance to use a pandemic app. Interestingly, this is in line with studies that showed significant differences in attitudes and behavior toward COVID-19 measures and policies between people living in East and West Germany [55,56]. For example, Fuest et al [55] tested the impact of pandemic information treatments on people residing in East and West Germany. They found that only West German citizens reacted significantly to the information, whereas East German citizens seemed far “less receptive to change their views based on information about economic or health aspects of the pandemic” [55]. This finding might also explain our result regarding the statistically lower rate of pandemic app uptake in East Germany. In general, differences in pandemic information and app uptake indicate that, even after 30 years of reunification, there are still experience-driven cultural and political differences toward governmental surveillance, tracing, and tracking measurements.

Regarding the factors of age and gender, other studies found that both were no or only weak predictors for pandemic app usage [20,23,26,32]. However, there are also studies indicating different tendencies for app adoption among different age groups [24,53,57]. Our results indicated no statistical correlation between personal affectedness by COVID-19 and pandemic app usage, which is contrary to previous studies that have suggested at least a weak significant impact of personal affection in terms of direct infection with COVID-19 on app adoption [20,32,58].

#### Two Patterns of Attitudes: Engagement Versus Privacy-Concerned Skepticism

Our study indicates a basic typology differing between users and nonusers of pandemic apps, which relies mainly on attitudinal features and less on sociodemographic factors. Type one—the data sharer—is characterized by high trust in state-funded research institutions and app providers, high willingness to share data, and seeing pandemic apps as useful for pandemic research as well as agreeing that there is a societal duty to share data to help with pandemic containment. The other type—the data-sharing skeptic—can be characterized by lower trust in state-funded app providers, decreased willingness for data sharing with research organizations, and considerably lower agreement with the view that using pandemics apps is a societal duty. These empirical findings can help to improve our understanding of who future app researchers would want to address. As problematized in other fields such as organ donation [59], technological skepticism among participants cannot sufficiently be explained by an information deficit. Hence, activities to increase the willingness to share data with research institutes might benefit from focusing on those willing to share or those who are yet undecided.

### Limitations

The findings in this report are subject to at least 3 limitations. First, with regard to inclusion criteria, the participants of the survey were all residents of Germany aged 18 years or older (which is also a common ethical-legal requirement for this type of survey) and accessible via a landline or mobile phone number, so no statements can be made for people younger than 18 years or people without any telephone connection or using call blockers. Since, for example, the national pandemic app (Corona Warn App) is available for teenagers aged 16 years and older, our sample of smartphone and app users is not exactly representative of all potential app users. However, since age was not a statistically significant factor for app uptake in our survey, the question arises whether including younger populations would have had statistically significant effects on public attitudes toward pandemic app usage. Regarding people using call blockers or people without landline or cell phone numbers, we could only speculate that these populations may have a rather skeptical attitude toward sharing app data.

Second, our decision to focus our statistical analysis on smartphone users (n=778) was based on considerations that eventually non-smartphone users may not have accurate conceptions and no concrete opinions about specific applications and app data, so answers by non-smartphone users about app details could have had a rather speculative character. The characteristics of the smartphone user sample slightly differ from the German population in 3 aspects: Smartphone users are somewhat younger (–3.35 years), slightly higher educated (4.4% more with A-Level or university degree), and slightly more often personally affected by current COVID-19 disease (3.7% more are affected). Therefore, the generalizability of the present results to older people and the overall German population is limited. Nonetheless, we consider our sample more informative for app developers and governance policies than surveys based on online panels involving a convenience sampling.

Finally, due to the time limitation for telephone surveys, we opted to not provide a definition for the “use” of smartphones and pandemic apps in our questionnaire (see Multimedia Appendix 1). As no specific criterion for usage was given, the interpretation of using a smartphone or using a pandemic app was made by the respondents. Time constraints also prevented us from asking participants about their usage of various, specific apps.

### Conclusions

The rapidly expanding field of apps in mHealth is very diverse with respect to architecture, features, and purposes. Smartphones users might be confused about different types of pandemic apps [17]. Our study focused on the potential for ROA—a relatively new field with high potential to become relevant for public health research and policy making on public health. Current app development is accompanied by governance policies and ELSI (Ethical, Legal, Social Issues) research. These frameworks already consider privacy and data safety perception of the broad population as key issues.

#### Social Implications for Governance of App Data

Our study indicated that trust in and trustworthiness of different app providers for data storage and app distribution, self-determination of data storage and transmission, and the social attitudes toward pandemic management are also crucial for such governance. Furthermore, lay-accessible information—also considering various sociocultural groups and different levels of eHealth literacy—should be part of future frameworks. Future research, (eg, on the incentivization of app adoption and data sharing or “data donation” [53,60]) might also evaluate to what extent trust and trustworthiness can be understood as an indirect incentive and what kind of incentivization is politically and ethically justifiable.

#### Ethical Implications for Pandemic App Development

In order not to gamble away the high willingness to share data via an app with state-funded research institutes, the life cycle of pandemic apps and all organizational providers involved in it should be made transparent.

From an ethical point of view, public-private partnerships for app development and app operation might be reconsidered because public and private app providers are perceived very differently among smartphone users. This applies all the more when it comes to public health emergencies such as pandemics when digital solutions are rapidly recommended to fix challenges in management and containment. At least, we assume, transparency of the engaged sectors and parties can help to engage as many citizens as necessary for valid ROA deployment.

## Appendix

Multimedia Appendix 1 Questionnaire translated into English.

Multimedia Appendix 2 Weighting model.

Multimedia Appendix 3 Flow chart: recruitment and sample.

Multimedia Appendix 4 Attitudes among people willing to share data (“data sharers,” n=653) for research via an app and people not willing to share data for research via an app (“non-data sharers,” n=125) among smartphone users (n=778).

Multimedia Appendix 5 Pandemic app use in West and East German Federal States.

## Abbreviations

API: application programming interface
BMBF: Bundesministerium für Bildung und Forschung
COMPASS: Coordination on mobile pandemic apps best practice and solution sharing
eHEALs: eHealth Literacy Scale
ELSI: Ethical, Legal, Social Issues
IT: information technology
mHealth: mobile health
NaFoUniMedCovid19: Forschungsnetzwerk der Universitätsmedizin zu COVID-19
ROA: research-orientated app
