# Supplementary material for: Attitudes Toward Mobile Apps for Pandemic Research Among Smartphone Users in Germany: National Survey
Source: JMIR Mhealth Uhealth. 2022 Jan 24;10(1):e31857. doi: 10.2196/31857 (PMC8822425; doi:10.2196/31857)
Supplement: Multimedia Appendix 1 [file mhealth_v10i1e31857_app1.pdf]

Questionnaire translated into English (original language: German)

Item nr.	GERMAN	ENGLISH[a]
**Intro**	**Vorstellung der Studie am Telefon**<br><br>Es geht um eine Umfrage zu einem aktuellen Thema. Wir befragen zu diesem Thema Personen aus der Bevölkerung in Deutschland ab 18 Jahren. Ihre Teilnahme ist natürlich freiwillig. Die Auswertung erfolgt anonym, also nicht in Verbindung mit Ihrem Namen oder Telefonnummer. Die Umfrage wird etwa 20 Minuten dauern. Möchten Sie an unserer Umfrage teilnehmen?	**Presentation of the study on the phone**<br><br>This is a survey on a current topic. We are surveying people from the population in Germany aged 18 and over on this topic. Your participation is of course voluntary. The evaluation will be anonymous, i.e., not in connection with your name or telephone number. The survey will take about 20 minutes. Would you like to take part in our survey?
**1) Nutzung eines Smartphones und von Pandemie-Apps**		**1) Current usage of smartphone and pandemic apps**
Q01	**Nutzen Sie ein Smartphone?**<br><br>• Ja. [1]<br>• Nein. [2]<br>• Weiß nicht, keine Angabe (nicht vorlesen)[3][b]	**Do you use a smartphone?**<br><br>• Yes. [1]<br>• No. [2]<br>• No specification. (not read out) [3][b]
	In den folgenden Fragen geht es um sogenannte Pandemie-Apps.<br><br>Wir verstehen unter Pandemie-Apps auf einem Smartphone installierte digitale Anwendungen zur Kontaktverfolgung oder	The following questions are about so-called pandemic apps.<br><br>We think of pandemic apps as digital applications installed on a smartphone for tracking contacts or providing information in the event of rapidly spreading

	Informationsbereitstellung bei sich schnell ausbreitenden Infektionskrankheiten wie der Grippe oder der aktuellen Corona-Pandemie. Ein Beispiel dafür ist die Corona-Warn-App der Bundesregierung.<br><br>(Nur auf Nachfrage: Eine App ist eine auf einem Smartphone installierte Software.)	infectious diseases such as influenza or the current Corona pandemic. One example is the federal government's Corona-Warn-App.<br><br>(Only if asked: An app is a software installed on a smartphone.)
Q02	[Wenn Q01 Ja oder weiß nicht, keine Angabe]<br>**Nutzen Sie zurzeit auf Ihrem Smartphone eine Pandemie-App?**<br><br>• Ja. [weiter mit Frage Q03a +Q04a] [1]<br>• Nein. [weiter mit Frage Q03b+Q04b] [2]<br>• Weiß nicht, keine Angabe. (INT.: NICHT vorlesen!) [weiter zu Q04b] [3]	[If Q01 Yes or don't know, not specified]<br>**Do you currently use a pandemic app on your smartphone?**<br><br>• Yes. [continue with question Q03a +Q04a] [1]<br>• No. [continue with question Q03b+Q04b] [2]<br>• Don't know, no indication. (not read out) [continue with Q04b] [3]
**2) Motivation, eine Pandemie- App (nicht) zu nutzen**		**2) Motivations for using or not using a pandemic app**
Q03a	[Wenn Q02 Ja]<br>**Warum nutzen Sie zurzeit eine Pandemie-App auf Ihrem Smartphone?** (Mehrfachnennung möglich)<br>[weiter mit Frage Q04a]<br><br>*ZUFALLSREIHENFOLGE CODES 1-5<br>• Ich leiste damit einen Beitrag zur Eindämmung der Infektionskrankheit. [1]<br>• Ich leiste damit einen Beitrag zur Erforschung der Infektionskrankheit. [2]<br>• Ich möchte mich damit vor einer Infektion schützen. [3]<br>• Ich möchte damit andere vor einer Infektion schützen. [4]<br>• Ich wurde von Menschen aus meinem persönlichen Umfeld gebeten, die App zu nutzen. [5]<br>• Anderer Grund. (INT.: NICHT vorlesen) *OPEN [6]	[If Q02 Yes]<br>**Why do you currently use a pandemic app on your smartphone?** (multiple answers possible) [continue with question Q04a]<br><br>RANDOM ORDER CODES 1-5<br>• I am contributing to the containment of the infectious disease. [1]<br>• I am using it to contribute to research into the infectious disease. [2]<br>• I want to use it to protect myself from infection. [3]<br>• I want to use it to protect others from infection. [4]<br>• I have been asked to use the app by people in my personal environment. [5]<br>• Other reason. (not read out) [6]<br>• Don't know, no indication. (not read out) [7]

	• Weiß nicht, keine Angabe. (INT.: NICHT vorlesen!) [7]	
Q03b	[Wenn Q02 Nein]<br>**Warum verwenden Sie zurzeit *keine* Pandemie-App auf Ihrem Smartphone?** (Mehrfachnennung möglich)<br>[weiter mit Frage Q04b]<br><br>*ZUFALLSREIHENFOLGE CODES 1-7<br>• Weil ich Zweifel habe, dass die App einen Nutzen für die Eindämmung der Infektionskrankheit hat. [1]<br>• Weil es mir zu mühsam ist, mich mit der App und den Daten zu beschäftigen. [2]<br>• Weil ich besorgt bin, dass Dritte meine Daten ohne meine Zustimmung nutzen. [3]<br>• Weil mir Hintergrundinformationen zur Datenerhebung und Datennutzung fehlen. [4]<br>• Weil ich mich dadurch von der App überwacht fühle. [5]<br>• Weil ich befürchte, dass sich durch die Nutzung der App die Akkulaufzeit meines Smartphones verringert. [6]<br>• Ich hatte in der Vergangenheit eine Pandemie-App genutzt, hatte aber technische Probleme damit. [7]<br>• Anderer Grund. (INT.: NICHT vorlesen) [8] *OPEN<br>• Weiß nicht, keine Angabe. (INT.: NICHT vorlesen!) [9]	[If Q02 No]<br>**Why do you currently NOT use a pandemic app on your smartphone?**<br>(multiple answers possible)<br>[continue with question Q04b]<br><br>RANDOM ORDER CODES 1-7<br>• Because I have doubts that the app is of any use in containing the infectious disease. [1]<br>• Because it is too tedious for me to deal with the app and the data. [2]<br>• Because I am concerned that third parties will use my data without my consent. [3]<br>• Because I lack background information on data collection and data use. [4]<br>• Because I feel monitored by the app. [5]<br>• Because I fear that using the app will reduce the battery life of my smartphone. [6]<br>• I had used a pandemic app in the past but had technical problems with it. [7]<br>• Other reason. (not read out) [8]<br>• Don't know, no indication. (not read out) [9]

Q04a	[Wenn Q02 ja]<br>**Beabsichtigen Sie, auch in Zukunft eine Pandemie-App auf Ihrem Smartphone zu nutzen?**<br>• Ja. [1]<br>• Nein. [2]<br>• Weiß nicht, keine Angabe. (INT.: NICHT vorlesen!) [3]	[If Q02 Yes]<br>**Do you intend to continue using a pandemic app on your smartphone in the future?**<br>• Yes. [1]<br>• No. [2]<br>• No specification. (not read out) [3]
Q04b	[Wenn Q02 nein]<br><br>**Beabsichtigen Sie in Zukunft, eine Pandemie-App auf Ihrem Smartphone zu nutzen?**<br><br>• Ja. [1]<br>• Nein. [2]<br>• Weiß nicht, keine Angabe. (INT.: NICHT vorlesen!) [3]	[If Q02 No]<br><br>**Do you intend to use a pandemic app on your smartphone in the future?**<br><br>• Yes. [1]<br>• No. [2]<br>• No specification. (not read aloud) [3]
**3) Vertrauen in App-Herausgeber und Datenspeicher**		**3) Trust in app distributors and data storage**
Q05	**Welche Herausgeber einer Pandemie-App halten Sie für vertrauenswürdig? (**Mehrfachnennung möglich)<br><br>*ZUFALLSREIHENFOLGE CODES 1-10<br>• Staatlich finanzierte Forschungseinrichtungen, z. B. Universitäten [1]<br>• Privat finanzierte Forschungseinrichtungen, z. B. forschende Biotechnologie- und Pharmaunternehmen [2]<br>• Bundes- oder Landesregierung [3]<br>• Regionales Gesundheitsamt [4]<br>• Staatlich finanzierte Kliniken [5]<br>• Privat finanzierte Kliniken [6]<br>• Gesetzliche Krankenkassen [7]<br>• Private Krankenversicherungen [8]	**Which distributor of a pandemic app do you consider trustworthy?**<br>(multiple answers possible)<br><br>RANDOM ORDER CODES 1-10<br>• State-funded research institutions, e.g. universities [1]<br>• Privately funded research institutions, e.g. research-based biotechnology and pharmaceutical companies [2]<br>• Federal or state government [3]<br>• Regional health authorities [4]<br>• State-funded clinics [5]<br>• Privately funded clinics [6]<br>• Statutory health insurers [7]<br>• Private health insurers [8]<br>• German software companies producing the app [9]

	• Deutsche Software-Unternehmen, die die App herstellen [9]<br>• Internationale Software-Unternehmen, die die App herstellen [10]<br>• Keinen davon. (INT.: NICHT vorlesen!) [98]<br>• Weiß nicht, keine Angabe. (INT.: NICHT vorlesen!) [99]	• International software companies producing the app [10]<br>• None of the above. (not read out) [98]<br>• No specification. (not read out) [99]
Q06	**Wo sollten die Daten, die eine Pandemie-App erhebt, Ihrer Meinung nach gespeichert werden?**<br>(Mehrfachnennung möglich)<br><br>*ZUFALLSREIHENFOLGE CODES 1-6<br>• Auf dem Smartphone selbst. [1]<br>• Bei einer gemeinnützigen Organisation, die als Treuhänder App-Daten verwaltet. [2]<br>• Bei einer staatlichen Forschungseinrichtung [3]<br>• Bei dem Software-Unternehmen, das die App herstellt. [4]<br>• Bei Bundesbehörden. [5]<br>• Nichts davon, woanders. (INT.: NICHT vorlesen!) [6] *OPEN<br>• Weiß nicht, keine Angabe. (INT.: NICHT vorlesen!) [9]	**Where do you think the data collected by a pandemic app should be stored?**<br>(multiple answers possible)<br><br>RANDOM ORDER CODES 1-6<br>• On the smartphone itself. [1]<br>• At a non-profit organisation that manages app data as a trustee. [2]<br>• At a government research institution. [3]<br>• At the software company that produces the app. [4]<br>• At federal agencies. [5]<br>• None of the above, anywhere else. (not read out) [6]<br>• Don't know, no indication. (not read out) [9]
**4) Bereitschaft zur Datenweitergabe an die Forschung und Einstellung gegenüber Datenhandhabe**		**4) Willingness to share coded data with research institutions using a pandemic app and attitudes toward data handling**
Q07	**Würden Sie die mithilfe einer Pandemie-App erhobenen Daten an staatliche Forschungsinstitute zur Verfügung stellen, wenn diese dann ausschließlich in kodierter Form weiterverwendet werden?**<br><br>Bei der Kodierung wird Ihren personenbezogenen Daten eine Nummer zugewiesen, sodass Ihre personenbezogenen Daten nicht direkt mit Ihnen in Verbindung gebracht werden können.<br><br>• Ja. [weiter mit Q08a und Q08b] [1]	**Would you provide data collected using a pandemic app to government research institutions if it was then only used in coded form?**<br><br>Coding is the process of assigning a number to your personal data so that your personal data cannot be directly linked to you.<br><br>• Yes. [1]<br>• No. [2]

	• Nein. [weiter mit Q09] [2]<br>• Weiß nicht, keine Angabe. (INT.: NICHT vorlesen!) [3]	• No specification. (not read out) [3]
Q08a	[Wenn Q07 Ja]<br>**Welche der mithilfe einer Pandemie-App erhobenen und kodierten Daten würden Sie der medizinischen Forschung zur Verfügung stellen?** (Mehrfachnennung möglich)<br>[weiter mit Q08b]<br><br>ZUFALLSREIHENFOLGE CODES 1-5<br>• Gesundheitsbezogene Daten, wie z. B. die eigene Körpertemperatur und Herzrate [1]<br>• Orts- und Bewegungsdaten. [2]<br>• Kontakte mit anderen Menschen. [3]<br>• Testergebnisse von Infektionstests. [4]<br>• Daten, die ein Fitness-Armband oder eine Fitness-Uhr erheben. [5]<br>• Andere Daten, die die App kontinuierlich ohne Ihr Zutun erhebt, z.B. die Umgebungstemperatur [6]<br>• Daten, die Sie selbst in die App eingetragen haben. [7]<br>• Nichts davon. (INT.: NICHT vorlesen!) [8]<br>• Weiß nicht, keine Angabe. (INT.: NICHT vorlesen!) [9]	[If Q07 Yes]<br><br>**Which of the data collected and coded using a pandemic app would you make available to medical research?**<br>(multiple answers possible)<br>[continue with question Q08b]<br><br>RANDOM ORDER CODES 1-5<br>• Health-related data, such as your own body temperature and heart rate. [1]<br>• Location and movement data. [2]<br>• Contacts with other people. [3]<br>• Test results from infection tests. [4]<br>• Data collected by a fitness wristband or fitness watch. [5]<br>• Other data that the app collects continuously without your intervention, e.g. ambient temperature. [6]<br>• Data that you have entered into the app yourself. [7]<br>• None of the above, anywhere else. (not read out) [8]<br>• Don't know, no indication. (not read out) [9]
Q08b	[Wenn Q07 Ja]<br>**Wäre es für Sie sehr wichtig, eher wichtig, nicht so wichtig oder überhaupt nicht wichtig, eine detaillierte Auflistung darüber zu erhalten, welche Forschungsinstitute die Daten Ihrer Pandemie-App auswerten?**<br>[weiter mit Q10]<br><br>• Sehr wichtig. [1]<br>• Eher wichtig. [2]<br>• Nicht so wichtig. [3]	[If Q07 Yes.]<br>**Would it be very important, rather important, not so important or not at all important for you to receive a detailed list of which research institutes analyse the data from your pandemic app?**<br>[continue with question Q10]<br><br>• Very important.<br>• Rather important. [1]<br>• Not so important. [2]<br>• Not at all important. [3]

	• Überhaupt nicht wichtig. [4]<br>• Weiß nicht, keine Angabe. (INT.: NICHT vorlesen!) [5]	• Not specified. [4]<br>• Don't know, no indication. (not read out) [5]
Q09	[Wenn Q07 Nein]<br>**Warum würden Sie die mithilfe einer Pandemie-App erhobenen und kodierten Daten *nicht* zur Verfügung stellen?**<br>(Mehrfachnennung möglich)<br><br>ZUFALLSREIHENFOLGE CODES 1-3<br>• Weil ich besorgt bin, dass Dritte meine Daten ohne meine Zustimmung nutzen. [1]<br>• Weil ich bezweifle, dass diese Daten der Forschung weiterhelfen. [2]<br>• Weil ich Sorge habe, dass die Daten im Rahmen eines sogenannten Datenlecks in die Öffentlichkeit gelangen. [3]<br>• Anderer Grund. (INT.: NICHT vorlesen!) [4] *OPEN<br>• Weiß nicht, keine Angabe. (INT.: NICHT vorlesen!) [5]	[If Q07 No]<br>**Why would you not provide the data collected and coded using a pandemic app?**<br>(multiple answers possible)<br><br>RANDOM ORDER CODES 1-3<br>• Because I am concerned that third parties will use my data without my consent. [1]<br>• Because I doubt that this data will help research. [2]<br>• Because I am worried that the data will be made public in a so-called data leak. [3]<br>• Other reason. (not read out) [4]<br>• Don't know, no indication. (not read out)
Q10	[Wenn Q07 Ja]<br>**Wie würden Sie die mithilfe einer Pandemie-App erhobenen Daten staatlichen Forschungsinstituten zur Verfügung stellen, damit diese dann kodiert weiterverwendet werden?**<br>(Mehrfachnennung möglich)<br><br>ZUFALLSREIHENFOLGE CODES 3-7<br>• indem das Smartphone die Daten automatisch an das Forschungsinstitut sendet. [1]<br>• indem ich die Daten in der App jedes Mal für das Forschungsinstitut freischalte, sodass sie vom Smartphone an das Forschungsinstitut gesendet werden [2]<br>• indem ich die Daten auf der Internetseite des Forschungsinstituts eintrage [3]	[If Q07 Yes]<br>**How would you make the data collected using a pandemic app available to government research institutes so that it can then be used in coded form?**<br>(multiple answers possible)<br><br>RANDOM ORDER CODES 3-7<br>• by having the smartphone automatically send the data to the research institute. [1]<br>• by enabling the data in the app each time for the research institute so that it is sent from the smartphone to the research institute. [2]<br>• by entering the data on the research institute's website. [3]<br>• by writing down the data in an e-mail and sending it to the research institute. [4]<br>• by writing down the data in an SMS and sending it to the research institute. [5]

	• indem ich die Daten in einer E-Mail notiere und an das Forschungsinstitut sende [4]<br>• indem ich die Daten in einer SMS notiere und an das Forschungsinstitut sende [5]<br>• indem ich bei einer Telefon-Hotline des Forschungsinstituts anrufe und die Daten dort angebe. [6]<br>• indem ich bei einer Video-Hotline des Forschungsinstituts anrufe und die Daten in einem Video-Gespräch angebe. [7]<br>• Nichts davon. (INT.: NICHT vorlesen!) [8]<br>• Weiß nicht, keine Angabe. (INT.: NICHT vorlesen!) [9]	• by calling a telephone hotline of the research institute and entering the data there. [6]<br>• by calling a video hotline of the research institute and providing the data in a video conversation. [7]<br>• None of the above, anywhere else. (not read aloud) [8]<br>• Don't know, no indication. (not read out) [9]
**5) Soziale Einstellung gegenüber App-Nutzung**		**5) Social attitude toward app use**
Q11	**Wie bewerten Sie folgende Aussage: „Die Nutzung von Pandemie-Apps ist eine gesellschaftliche Verpflichtung.“**<br><br>• Stimme voll und ganz zu. [1]<br>• Stimme eher zu. [2]<br>• Stimme weder zu noch nicht zu. [3]<br>• Stimme eher nicht zu. [4]<br>• Stimme überhaupt nicht zu. [5]<br>• Weiß nicht, keine Angabe (INT.: NICHT vorlesen!) [6]	**How do you rate the following statement: "The use of pandemic apps is a social obligation."**<br><br>• Agree fully. [1]<br>• Rather agree. [2]<br>• Neither agree nor disagree. [3]<br>• Completely disagree. [4]<br>• Do not agree at all. [5]<br>• Don't know, no indication (not read out). [6]
Q12	**Welche Quellen nutzen Sie, um sich über das neuartige Coronavirus zu informieren?**<br>(Mehrfachnennung möglich)<br><br>ZUFALLSREIHENFOLGE CODES 1-7<br>• Fernsehen [1]<br>• Radio [2]<br>• Internet allgemein [3]<br>• Gedruckte Zeitungen oder Zeitschriften [4]	**Which sources do you use to inform yourself about the novel coronavirus?**<br>(multiple answers possible)<br><br>RANDOM ORDER CODES 1-7<br>• Television [1]<br>• Radio [2]<br>• Internet in general [3]<br>• Print newspaper or magazines [4]<br>• Social networks on the Internet, e.g. Facebook or Twitter [5]

	• soziale Netzwerke im Internet, z. B. Facebook oder Twitter [5]<br>• Gespräche mit Freunden oder Bekannten [6]<br>• Gespräche mit Kollegen oder im beruflichen Umfeld [7]<br>• Andere Quellen (INT.: NICHT vorlesen!) [8] *OPEN<br>• Keine Angabe. (INT.: NICHT vorlesen!) [9]	• Conversations with friends or acquaintances [6]<br>• Conversations with colleagues or in a professional environment [7]<br>• Other sources. (not read out). [8]
**6) Soziodemografische und persönliche Merkmale**		**6) Socio-demographic and personal characteristics**
Q13	**Welches Geschlecht haben Sie?**<br><br>• Weiblich [1]<br>• Männlich [2]<br>• Divers [3]<br>• keine Angabe (INT.: NICHT vorlesen!) [4]	**What gender are you?**<br><br>• Female [1]<br>• Male [2]<br>• Gender neutral [3]<br>• Not specified [4]
Q14	**Darf ich fragen, wie alt Sie sind?**<br><br>- - (INT.: Bitte Alter in Jahren eingeben.)<br>_____________ Jahre<br>• keine Angabe (INT.: NICHT vorlesen!) [999]	**May I ask how old you are?**<br><br>- (INT.: Please enter age in years.)<br>_____________ years<br>- not specified (not read out) [999]
Q15	**Welches ist Ihr höchster Schul- oder Studienabschluss?**<br><br>• Kein Schulabschluss [1]<br>• Volks- oder Hauptschulabschluss [2]<br>• Mittlere Reife oder Abschluss der Polytechnischen Oberschule [3]<br>• Abitur, Fachhochschulreife [4]<br>• abgeschlossenes Studium: Bachelor [5]<br>• abgeschlossenes Studium: Master/Magister/Diplom/Staatsexamen [6]<br>• Promotion [7]<br>• Noch in Schulausbildung [8]	**What is your highest school or university degree?**<br><br>• No school-leaving qualification [1]<br>• Elementary or secondary school leaving certificate [2]<br>• Secondary school leaving certificate or polytechnic secondary school leaving certificate [3]<br>• A-levels, technical college entrance qualification [4]<br>• Completed studies: Bachelor's Degree [5]<br>• Completed studies: Master's Degree/Magister/Diploma/State examination [6]<br>• Doctorate [7]<br>• Still in school [8]

	• Keine Angabe. (INT.: NICHT vorlesen!) [9]	• Not specified [9]
Q16	**Sind Sie oder Ihre Eltern nach Deutschland eingewandert ODER hatten Sie oder Ihre Eltern bei der Geburt eine ausländische Staatsangehörigkeit?**<br><br>• Ja. [1]<br>• Nein. [2]<br>• Keine Angabe. (INT.: NICHT vorlesen!) [3]	**Did you or your parents immigrate to Germany OR did you or your parents have a foreign nationality at birth?**<br><br>• Yes. [1]<br>• No. [2]<br>• No specification. (not read out) [3]
Q17	**Gibt es in Ihrem persönlichen Umfeld eine Person, die mit dem neuartigen Coronavirus infiziert ist oder war, inkl. Sie selbst?**<br><br>• Ja. [1]<br>• Nein. [2]<br>• Weiß nicht, keine Angabe. (INT.: NICHT vorlesen!) [3]	**Is there anyone in your personal environment who is or has been infected with the novel coronavirus, including yourself?**<br><br>• Yes. [1]<br>• No. [2]<br>• No specification. (not read out) [3]

[a] We acknowledge Nicole Chanady, KERN AG, Sprachendienste (Würzburg, Germany), for the English translation.

[b] Internal Instructions for the interviewer.
