# Supplementary material for: Attitudes Toward Mobile Apps for Pandemic Research Among Smartphone Users in Germany: National Survey
Source: JMIR Mhealth Uhealth. 2022 Jan 24;10(1):e31857. doi: 10.2196/31857 (PMC8822425; doi:10.2196/31857)
Supplement: Multimedia Appendix 2 [file mhealth_v10i1e31857_app2.pdf]

Multimedia Appendix 2

Weighting Model

In order to adjust for differing selection probabilities in the combined landline and cell-phone sample, the following design weighting model was applied†:

1. Step

Inclusion probability of a target person

m^L	Sample size of landline numbers *(m^F)*
M^L	Frame size of valid landline numbers *(M^F)*
k_i^L	Count of landline numbers allowing access to target person i *(k_i^F)*
m^C	Sample size of cell-phone numbers *(m^C)*
M^C	Frame size of valid cell-phone numbers *(M^C)*
k_i^C	Count of cell-phone numbers allowing access to target person i *(k_i^C)*
Z_{HH}	Number of target persons in the household to which target person i belongs *(Z_i)*

Design weights: *$1/\pi_i$*

$$\pi_i \approx k_i^F \frac{m^F}{M^F} \cdot \frac{1}{z_i} + k_i^C \frac{m^C}{M^C}, i = 1, \dots, N$$

$m^{F/C}$	Anzahl Nummern in der Stichprobe (Festnetz / Mobilfunk)
$M^{F/C}$	Anzahl gültiger Rufnummern insg. (Festnetz / Mobilfunk)
$k_i^{F/C}$	Anzahl der Rufnummern (Festnetz / Mobilfunk) über die der Haushalt bzw. die Person erreicht werden kann
z_i	Anzahl Zielpersonen im Haushalt

The following variables are based on items asked in the questionnaire:

$k_{i\,L}$	Count of landline numbers allowing access to target person i *(k_i^F)*
k_i^C	Count of cell-phone numbers allowing access to target person i *(k_i^C)*
Z_{HH}	Number of target persons in the household to which target person i belongs *(Z_i)*

2. Step

In a second step, the structural discrepancies commonly existing within random samples (over- or under-representation of specific socio-demographic groups) were compensated by redressement weighting. The sample has been weighted in terms of the characteristic's region, city size, age, gender, and education:

- Federal state
- BIK community size
- Region (North/West/South/East) x Age groups
- Region (North/West/South/East) x Gender
- Region (North/West/South/East) x Level of education
- Age groups x Gender
- Level of education

The product of design weight x redressement weight is included in the data set in the form of the variable "weight".

Weighting ensures that the sample, forming the basis of the analysis, corresponds to the structure of the basis population as for its composition.
Hence the data collected can be generalized for the basic population within the limits of the statistical tolerances.

† *We thank Kantar GmbH for providing the details of the applied weighting model.*
