# Supplementary material for: Attitudes Toward Mobile Apps for Pandemic Research Among Smartphone Users in Germany: National Survey
Source: JMIR Mhealth Uhealth. 2022 Jan 24;10(1):e31857. doi: 10.2196/31857 (PMC8822425; doi:10.2196/31857)

Multimedia Appendix 3

Flow chart: recruitment and sample[a]

Aim: Representative national telephone-based survey based on a sample of 1000 interviews.

[a] Data for recruitment provided by Kantar GmbH

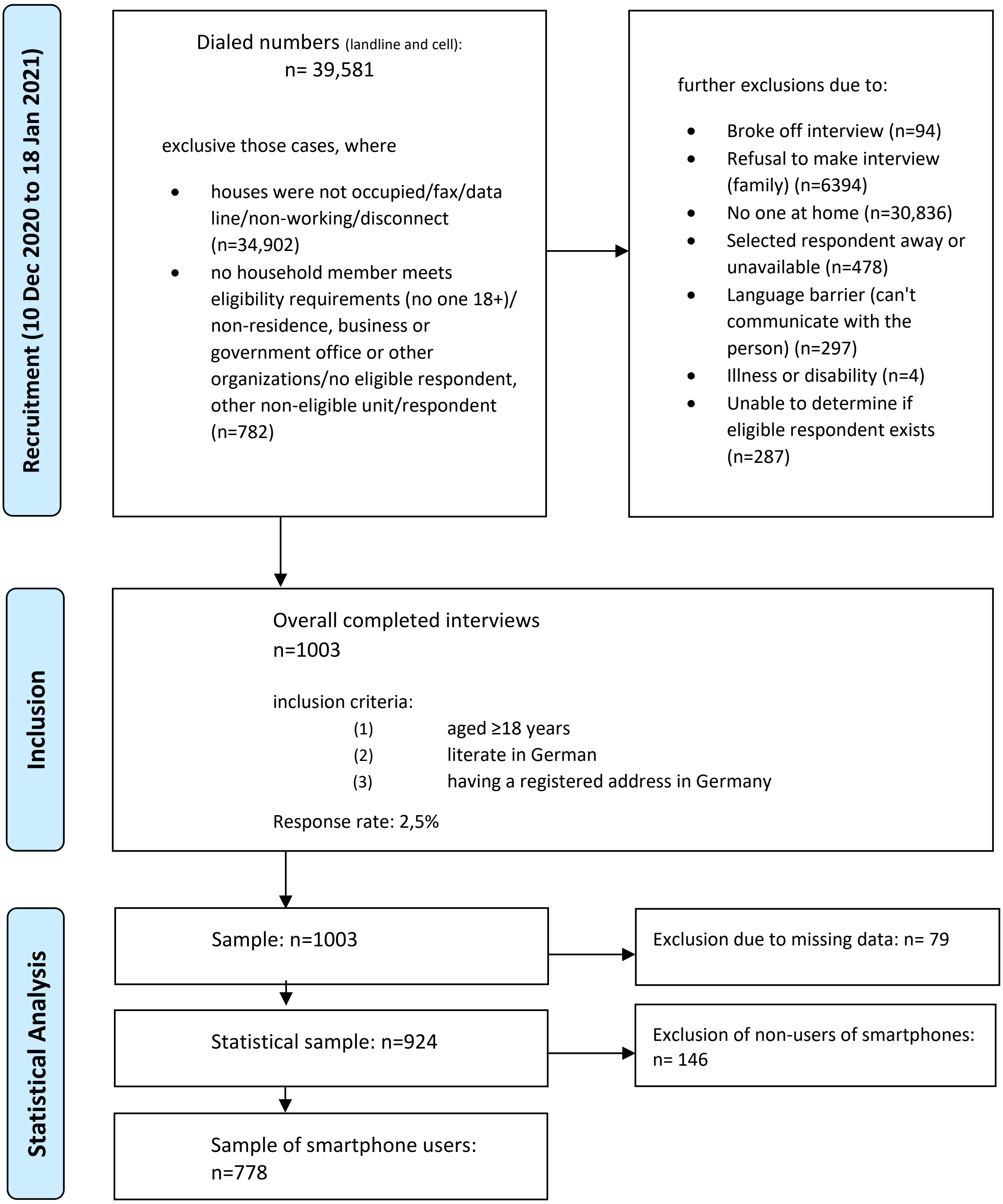

Supplement: Multimedia Appendix 3 [file mhealth_v10i1e31857_app3.pdf]
