# Supplementary material for: Attitudes Toward Mobile Apps for Pandemic Research Among Smartphone Users in Germany: National Survey
Source: JMIR Mhealth Uhealth. 2022 Jan 24;10(1):e31857. doi: 10.2196/31857 (PMC8822425; doi:10.2196/31857)
Supplement: Multimedia Appendix 4 [file mhealth_v10i1e31857_app4.pdf]

Multimedia Appendix 4

Figure 1. Attitudes among people willing for data sharing ("data sharers", n=653) with research via an app and people not willing for data sharing with research via an app ("non-data sharers", n=125) among smartphone users (n=778).

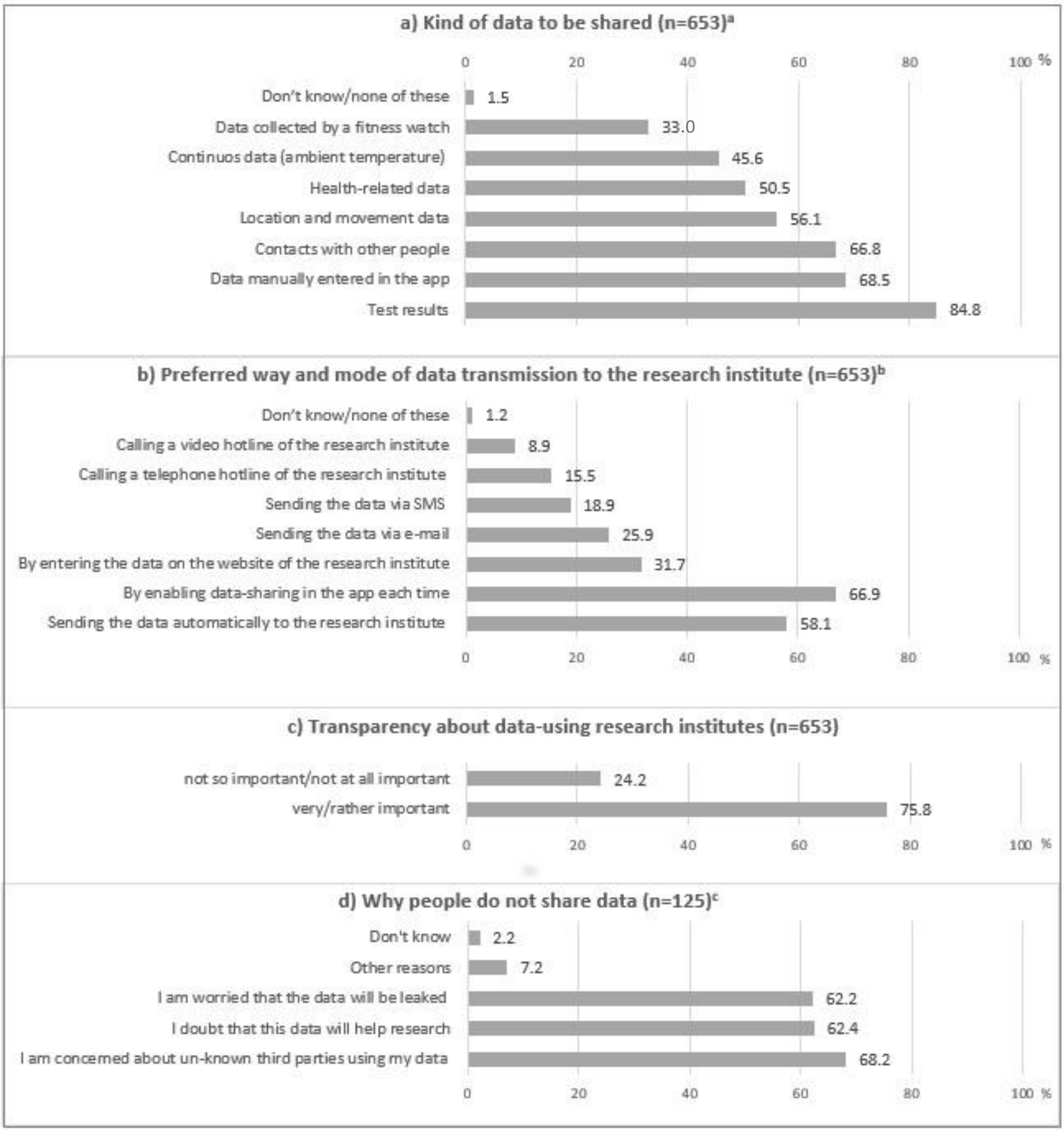

[a] Multiple answers were possible.
[b] Multiple answers were possible.
[C] Multiple answers were possible.
