# Supplementary material for: Attitudes Toward Mobile Apps for Pandemic Research Among Smartphone Users in Germany: National Survey
Source: JMIR Mhealth Uhealth. 2022 Jan 24;10(1):e31857. doi: 10.2196/31857 (PMC8822425; doi:10.2196/31857)

Multimedia Appendix 5: **Pandemic App Use in West and East German Federal States**

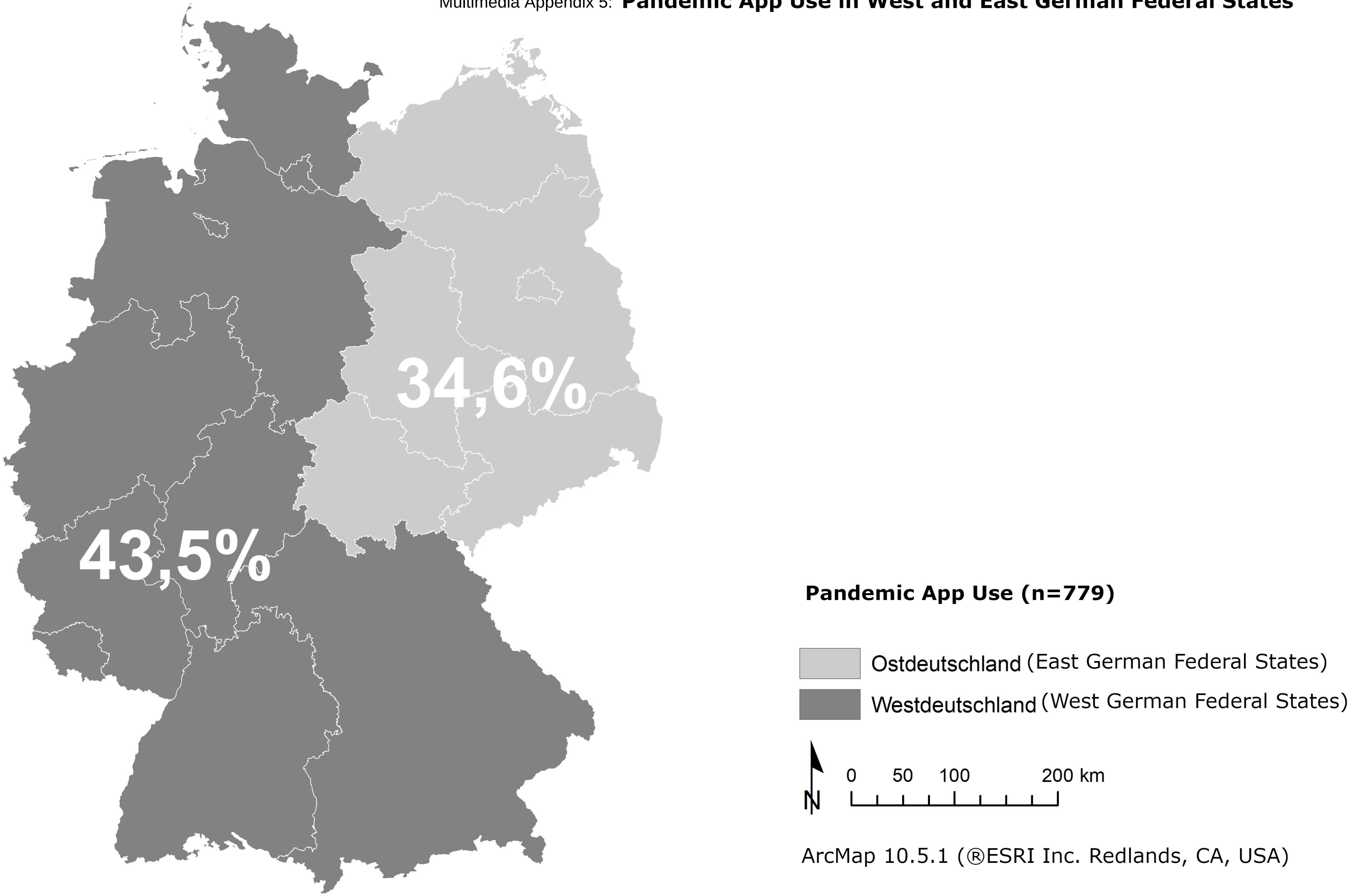

Supplement: Multimedia Appendix 5 [file mhealth_v10i1e31857_app5.pdf]
